# Genetic insights into bacterial wilt resistance using genomic prediction and association mapping in blueberry

**DOI:** 10.1002/tpg2.70283

**Published:** 2026-08-20

**Authors:** Lushan Ghimire, Norma Flor, Paul Adunola, Philip F. Harmon, Felix Enciso‐Rodriguez, Juliana Benevenuto, Luis Felipe V. Ferrão, Patricio R. Munoz

**Affiliations:** ^1^ Blueberry Breeding and Genomics Lab, Horticultural Sciences Department University of Florida Gainesville Florida USA; ^2^ Plant Pathology Department University of Florida Gainesville Florida USA; ^3^ Plant Breeding Graduate Program University of Florida Gainesville Florida USA

## Abstract

Bacterial wilt, caused by *Ralstonia* spp., poses a major threat to blueberry (*Vaccinium corymbosum*) production due to its persistence and rapid spread through soil and infected stock, highlighting the need for genetic insights to guide breeding strategies. This study investigated the genetic basis of bacterial wilt resistance in blueberry using a genome‐wide association study (GWAS) across two populations comprising 401 advanced selections from the University of Florida Blueberry Breeding and Genomics Program. A high‐throughput screening assay was developed to evaluate southern highbush blueberry responses to bacterial wilt based on leaf wilting severity and stem necrosis. Capture sequencing identified 38,379 single‐nucleotide polymorphisms. Moderate narrow‐sense heritability estimates were observed for leaf severity (0.26) and stem necrosis (0.20), and GWAS identified five small‐effect quantitative trait loci on chromosomes 1, 2, 5, and 11, each explaining 4.0%–7.4% of the phenotypic variance. Candidate gene analysis revealed putative pentatricopeptide repeat (PPR), serine/threonine protein kinase, and MYB‐related proteins for leaf severity, and *Mlo* genes and polysaccharide biosynthesis genes for stem necrosis. Genomic selection (GS) analyses demonstrated potential for improving bacterial wilt resistance, with the GS *de novo* GWAS approach achieving the highest predictive ability by leveraging two key markers on chromosomes 1 and 11. These results elucidate the genetic architecture of bacterial wilt resistance in blueberries and provide resources for molecular breeding strategies to enhance resistance and ensure sustainable production.

## Core Ideas

- Genome‐wide association and genomic prediction analyses reveal the genetic basis of bacterial wilt resistance in blueberry.
- Statistical and machine learning models show differing abilities to predict disease resistance traits.
- Genomic‐enabled prediction provides a practical framework for improving selection decisions in blueberry breeding.

## Plain Language Summary

Bacterial wilt is a destructive disease of blueberry that spreads through soil and infected planting material, making it difficult to manage once established. One of the best long‐term solutions is to develop blueberry varieties that are naturally resistant to disease. In this study, we evaluated more than 400 blueberry selections to better understand the genetic factors involved in bacterial wilt resistance. We developed a rapid screening method to measure disease symptoms, allowing many plants to be tested efficiently. We found that resistance differs among plants and is controlled by several small genetic regions linked to plant defense and cell wall strength. Using this genetic information, we showed that resistance can be predicted early, even before plants are fully tested in the field. These findings can help breeders select resistant plants earlier and more reliably, supporting the development of resistant blueberry varieties and more sustainable production systems.

**Abbreviations**
BGLR: Bayesian generalized linear regression
BLUE: best linear unbiased estimate
CV: cross‐validation
GBLUP: genomic best linear unbiased prediction
GS: genomic selection
GWAS: genome‐wide association study
LD: linkage disequilibrium
MAS: marker‐assisted selection
MCMC: Markov chain Monte Carlo
ML: machine learning
PA: predictive ability
QTL: quantitative trait locus
RF: random forest
ROS: reactive oxygen species
RSSC: *Ralstonia solanacearum* species complex
SHB: southern highbush blueberry
SNP: single‐nucleotide polymorphism
SVM: support vector machine
TZC: triphenyl tetrazolium chloride
XGB: extreme gradient boosting

## 1 INTRODUCTION

*Ralstonia solanacearum*, a species complex (RSSC) with high genetic diversity (Vailleau & Genin, 2023), affects over 200 plant species and induces various disease symptoms (Elphinstone, 2005). Its extensive host range, genetic variability, wide geographical distribution, and severe disease impact have led to its recognition as one of the top 10 plant pathogenic bacteria worldwide (Mansfield et al., 2012). In the southern United States, this pathogen has caused significant losses in crops such as tobacco, potato, tomato, ornamentals, and blueberries (Norman et al., 2020). RSSC can persist in damp soil, entering plants through wounds, natural openings, or roots, subsequently colonizing xylem vessels and causing wilting symptoms (Bocsanczy et al., 2017; Buddenhagen & Kelman, 1964).

The RSSC was reported to cause bacterial wilt disease in southern highbush blueberries (SHBs, *Vaccinium corymbosum* and hybrids) (Norman et al., 2018). Three distinct populations of *Ralstonia* were identified in Florida using genomics and proteomics approaches: two populations classified under *R. solanacearum*, phylotype II sequevar 7 and 38, and one under *Ralstonia pseudosolanacearum*, phylotype I sequevar 13 (Bocsanczy et al., 2019). Bocsanczy et al. (2022) reported that the *R. pseudosolanacearum* population has become the predominant pathogenic strain affecting blueberry production.

Bacterial wilt on highbush blueberries was also reported in New Jersey in 2012 (Harmon et al., 2016) and in Georgia in 2023 (Oliver et al., 2023). With the rapid expansion of the blueberry industry into areas harboring endemic pathogen populations, multiple wilt outbreaks are possible (Norman et al., 2018). In the 2023 Annual Blueberry Season Survey, bacterial wilt was highlighted as the second most concerning disease among Florida blueberry growers, following leaf rust (Phillips, 2023). Affected plants exhibit wilt‐like symptoms, including marginal leaf burn, bronzing of leaves, necrotic leaves, drought stress, and other stress‐like symptoms such as stem blight and mottled discoloration (ranging from light brown to silvery purple blotches) on the crowns (Norman et al., 2018). Bacterial ooze can be observed when diseased wood chips are floated in water (Harmon et al., 2016). The disease spreads easily through practices like using recycled irrigation water and sharing pruning equipment between infected and healthy plants. It can persist in soil for several years, spreading within and across blueberry rows, leading to extensive circular patches of wilted bushes (Harmon et al., 2016). Infected plants can also remain symptomless in unfavorable conditions, facilitating rapid disease dissemination (across continents and countries) through the sale of asymptomatic infected stock (Norman et al., 2020).

Current disease management strategies include preventing microbe infiltration into the farm, soil treatments, and burning or deep‐burying infected debris (Harmon et al., 2016). The most economically viable and environmentally sustainable approach to mitigating losses caused by this disease would be the adoption of bacterial‐wilt‐resistant blueberry cultivars on farms.

Traditional breeding methods for developing disease‐resistant cultivars are laborious and resource‐intensive (Slater et al., 2013). However, the use of molecular breeding tools could accelerate trait selection in breeding programs. Advances in sequencing and automation technologies have significantly reduced genotyping costs, enabling the use of molecular markers. For a long time, these markers, combined with quantitative trait locus (QTL) mapping, have played a crucial role in pinpointing genomic regions linked to specific traits. In contrast to QTL mapping, genome‐wide association studies (GWASs) enhance mapping resolution by utilizing diverse populations with reduced levels of linkage disequilibrium (LD) and increased marker density. This approach effectively narrows down genomic regions, facilitating the identification of potential candidate genes and causal polymorphisms (Ferrão et al., 2020; Korte & Farlow, 2013). In blueberries, GWASs have successfully elucidated the genetic basis of various traits, including fruit quality, off‐season flowering, flavor‐related volatiles (da Silva et al., 2024; Ferrão et al., 2018, 2020), and diseases such as anthracnose (Ghimire et al., 2025; Jacobs et al., 2023) and gray mold (Ghimire et al., 2026). On the other hand, while molecular breeding efforts for resistance to bacterial wilt have been reported in several plant species such as tomato (Abebe et al., 2020), potato (Gao et al., 2000), eggplant (Pandiyaraj et al., 2019), and pepper (Thakur et al., 2014), no studies have been performed in blueberries so far. This underscores the need to initiate breeding of bacterial‐wilt‐resistant blueberry cultivars using molecular markers.

In this study, we developed a high‐throughput screening assay and evaluated the responses of advanced selections of SHBs to bacterial wilt. We then employed GWAS to uncover genomic regions associated with resistance to this disease. Our study revealed significant genetic and phenotypic variability across two breeding populations, identifying marker associations and potential candidate genes linked to leaf severity and stem necrosis due to bacterial wilt. Moreover, the feasibility of using genomic prediction to select bacterial‐wilt‐resistant blueberries in the breeding program was also assessed. These findings provide valuable insights into the genetic foundations of resistance in a breeding population, which is essential for optimizing molecular strategies to enhance bacterial wilt resistance in blueberries.

## 2 MATERIALS AND METHODS

### 2.1 Plant material

**FIGURE 1:**
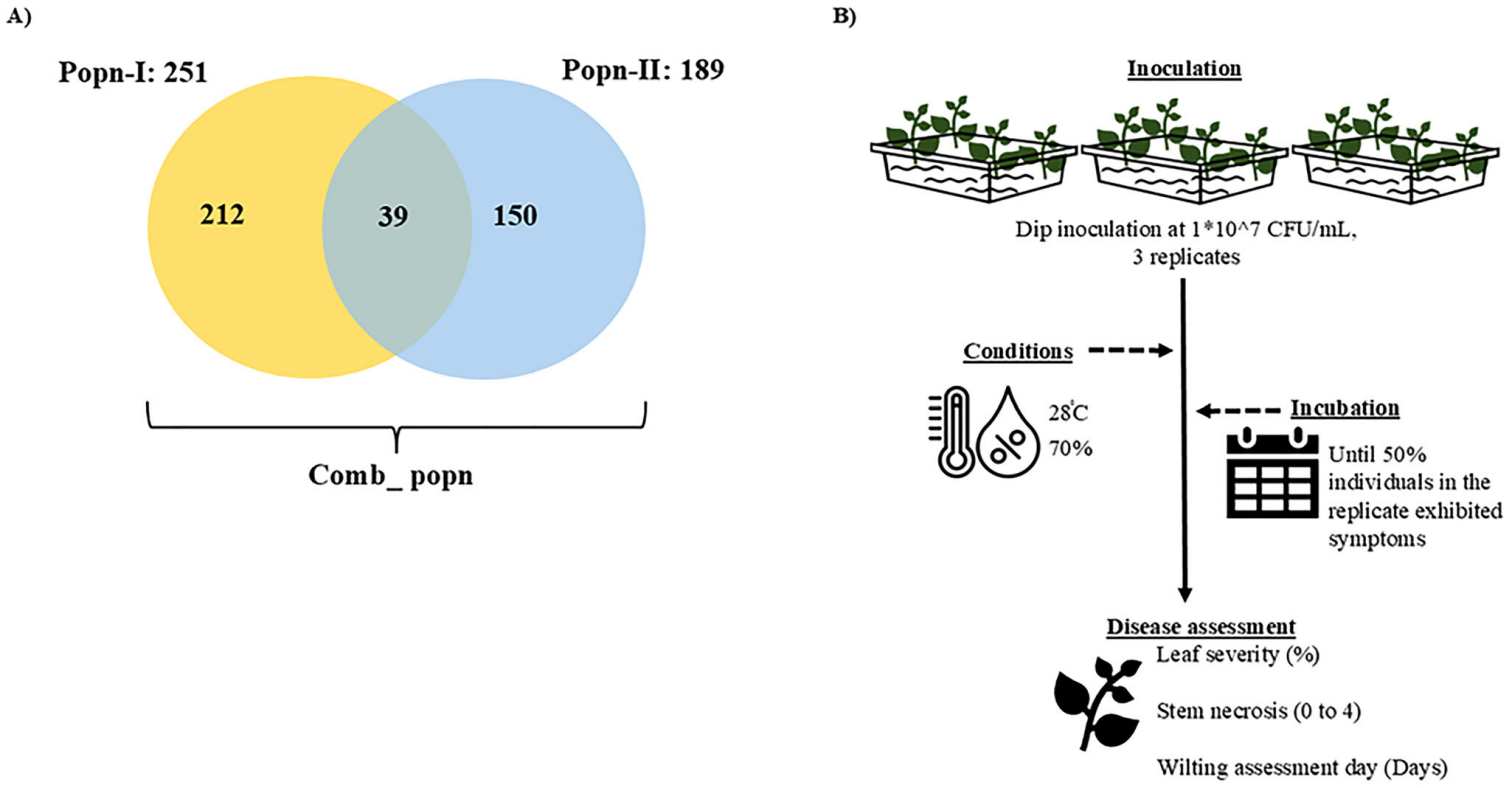
Details of phenotyping of blueberries for resistance to bacterial wilt. (A) Venn diagram showing the number of individuals within each of the two populations used in the study, including overlapping individuals. (B) Screening protocol developed for bacterial wilt resistance assessment in a breeding population level.

Phenotyping evaluations were conducted using two SHB populations, comprising a total of 401 advanced clonal selections from a common breeding pool within the University of Florida Blueberry Breeding Program. Although all selections share pedigree origins and exhibit varying degrees of relatedness, the two populations were drawn from successive selection cycles, each with different parental combinations, and thus capture complementary genetic diversity. The first population (Popn‐I, *n* = 251) was evaluated for bacterial wilt resistance in September 2021, and the second population (Popn‐II, *n* = 189) was similarly phenotyped in August 2023, of which 39 genotypes overlapped with Popn‐I and served as checks to assess inter‐year variation (Figure 1A). Plant material was sourced from test plots in Waldo, FL, managed under standard commercial blueberry production practices. For every genotype, six softwood cuttings of approximately 35–40 cm in length were collected and stored at 0°C for 24 h prior to inoculation.

**Core Ideas**

- Genome‐wide association and genomic prediction analyses reveal the genetic basis of bacterial wilt resistance in blueberry.
- Statistical and machine learning models show differing abilities to predict disease resistance traits.
- Genomic‐enabled prediction provides a practical framework for improving selection decisions in blueberry breeding.

### 2.2 Bacterial isolate and inoculum preparation

The inoculum for both populations was prepared using *R. pseudosolanacearum* (isolate P824, G16‐1529, phylotype I, sequevar 13), which was first isolated by Dr. Philip Harmon in 2016 from naturally infected stems of the Arcadia cultivar at a commercial blueberry farm in Central Florida (Bocsanczy et al., 2019) and is now recognized as the region's predominant blueberry‐infecting lineage (Bocsanczy et al., 2022; Conner et al., 2022). The bacterial isolate was first activated on triphenyl tetrazolium chloride (TZC) media plates and incubated under dark conditions between 28°C and 30°C for 48 h. TZC media separated the non‐virulent and virulent colonies, where dark red colonies were non‐virulent and creamy white colonies with pink centers were virulent (Rudrappa et al., 2016). Only freshly grown virulent bacterial colonies were used to streak onto additional TZC media plates and incubated under the conditions described above. Finally, bacterial colonies were resuspended to a final concentration of 1 × 10^7^ CFU (colony‐forming unit)/mL, measured using a spectrophotometer at 280 nm for optical density. Twenty liters of the same bacterial suspension were added to a plastic trough (Model: G5435768, Bayhead Products, Zoro) measuring 144.8 cm × 45.7 cm × 19.7 cm. Similar inocula were prepared for two additional plastic troughs using the aforementioned protocol.

### 2.3 Inoculation procedure

For each genotype, two randomly selected softwood cuttings were bundled together to form a replicate bundle. Three replicate bundles were set up per genotype. For inoculation, all softwood cuttings were cut at the base with pruning shears and dipped (dip inoculation method) in inoculum‐filled plastic troughs until the end of the experiment (Figure S1). Each trough constituted a randomized complete block, with one replicate (comprising two cuttings) of each genotype. Stem cuttings of cultivars Emerald, Indigocrisp, Arcadia, and Ochlockonee were also included as negative controls and were dipped in deionized water instead of the inoculum. The experimental setup was maintained within a walk‐in growth chamber at 60%–70% relative humidity and a temperature of 28°C (Figure 1B, Figure S1). The water level in the plastic troughs was kept constant by adding deionized water to each trough every 2–3 days. The genotypes were monitored daily during incubation until 50% of the genotypes in each replicate exhibited wilting symptoms (Figure 1B). Consequently, some genotypes remained in the inoculum longer than others, an aspect that was accounted for in the analysis by recording “wilting assessment days,” details of which are discussed later in this paper. For re‐isolating the bacterium during the incubation, inoculated leaf and stem tissues (both symptomatic and asymptomatic) were collected from the top one‐third region of the cuttings of six randomly chosen genotypes from each replication in addition to the control samples. This procedure was performed to confirm bacterial translocation to the upper parts of the cuttings. The excised tissue underwent surface sterilization with a 10% bleach solution for 1 min, followed by three 1‐min water rinses. Subsequently, the tissue was immersed in sterile deionized water for approximately 10 min, and the resulting extract was plated onto TZC media plates and incubated as described previously. Typical bacterial colonies of *R. pseudosolanacearum* resembling the original reference isolate were observed in 100% of the inoculated samples (both symptomatic and asymptomatic), but not in the control samples.

### 2.4 Disease phenotyping

To evaluate the disease response of the genotypes, severity of symptoms in leaves that includes wilting, desiccation, water‐soaked lesions, and necrosis (leaf severity) and extent of necrosis in stems (stem necrosis) were assessed. Comprehensive images covering all detached leaves and stem tissues for each genotype were captured. Images were visually assessed to estimate the severity percentage of the total leaf area affected by wilt‐like symptoms, and the two stems of each genotype were independently rated on a scale of 0–4 for necrosis symptoms. The scores ranged from 0 to 4, where 0: none, 1: <25%, 2: 25%–50%, 3: 50%–75%, 4: >75% of necrosis present on the stems. The scores from two stems were then averaged for analysis.

Due to the large number of genotypes being screened, assessing disease severity for all genotypes in three replicates on the same day posed logistical challenges. Consequently, some genotypes would have prolonged exposure to the inoculum compared to others, potentially affecting disease severity ratings. To address this variability, the “wilting assessment day” was accounted. The wilting assessment day represents the postinoculation day when the disease severity in the given genotype was assessed. Incorporating the wilting assessment day as a covariate in subsequent analyses would enable us to adjust ratings based on exposure duration, thereby improving the accuracy and reliability of disease severity evaluations.

### 2.5 Genotypic data

Fresh, juvenile leaves were collected from each genotype for subsequent genotyping processes. DNA extraction and genotyping were carried out at RAPiD Genomics using the Capture‐Seq method. A genome‐wide panel comprising 10,000 biotinylated probes spanning around 120‐mers was used for targeted capture. This was followed by high‐throughput sequencing on the Illumina HiSeq2000 platform using 150‐cycle paired‐end runs.

Single‐nucleotide polymorphism (SNP) calling and filtering procedures adhered to the methodology by Ferrão et al. (2021). Initially, raw reads were subjected to quality‐based filtering and trimming. The filtered reads were then aligned to the largest haploid scaffold set derived from the *V. corymbosum* ‘Draper’ genome (Colle et al., 2019) using Mosaik v.2.2.3 (W.‐P. Lee et al., 2014). Subsequently, SNPs were called with the 10,000 probe positions as targets using FreeBayes v.1.3.2 (Garrison & Marth, in press). Criteria for SNP filtering included a minimum mapping quality of 10, inclusion of only biallelic loci, a maximum missing data threshold of 50%, and a minor allele frequency threshold of 0.01. Sequencing read counts per allele and individuals were extracted from the variant call file using Vcftools v. 0.1.16 (Danecek et al., 2011). Allele dosages were obtained based on the read counts using the updog package v.2.1.0 in R, as described by Gerard et al. (2018). Only SNPs accurately genotyped in 95% of the individuals were retained, utilizing the “prop_mis” function. The ploidy parameterization was established by encoding the dosage of the alternative allele (B) in relation to the reference allele (A) as follows: 0 (AAAA), 1 (AAAB), 2 (AABB), 3 (ABBB), and 4 (BBBB) (Rosyara et al., 2016).

### 2.6 Phenotypic analysis

The datasets from both populations (Popn‐I and Popn‐II) were combined (Comb_popn), separately for leaf and stem symptoms, and analyzed. The wilting assessment day was employed as a covariate in the model, and the best linear unbiased estimates (BLUEs) were estimated for leaf severity and stem necrosis. Notably, for BLUEs estimation in Comb_popn, a one‐stage correction was applied using the overlapping 39 genotypes as checks to correct for year‐effects. Thus, adjusted means for each genotype were obtained using the following linear model:

$$\;y_{ijk}=\mu +B_{i}+R/B_{ij}+G_{ijk}+W_{ijk}+e_{ijk}$$

where 𝑦_𝑖𝑗𝑘_ is the phenotype of the disease response (leaf severity or stem necrosis) of genotype *i* in year *j* and replicate *k*; 𝜇 is the overall mean; 𝐵_i_ is the fixed year effect; 𝑅/𝐵_𝑖𝑗_ is the fixed replicate effect under each year; 𝐺_𝑖𝑗𝑘_ is the genotypic effect of individual *i* at year *j* and replicate *k*; 𝑊_𝑖𝑗𝑘_ is the wilting assessment day of individual *i* at year *j* and replicate *k*; and 𝑒_𝑖𝑗𝑘_ is the random residual effect assumed to be independent and normally distributed (𝑒_𝑖𝑗𝑘_∼𝑁(0, 𝜎_e_^2^)), where 𝜎_e_^2^ is the residual variance. For the genotypic effect, the experiment was designed as an augmented block design with common checks connecting both years. Thus, the genotypic effect was separated into two groups, where 𝑔 is the fixed effect for the regular individuals and 𝑐 is the fixed effect associated to the checks. The BLUEs of each genotype were used as the response variable in subsequent analyses. Similarly, the BLUEs for each population were also estimated separately using the same model, considering *Bi *= 0 for genomic selection (GS) scenarios.

Furthermore, heritability estimates were computed to quantify the proportion of additive and nonadditive genetic variation attributed to genetic factors by considering genotypes as random effects and incorporating genomic information into the relationship matrix:

$$\;y^{*}=1\mu +Z_{a}a+Z_{d}d+e$$

where y^*^ is the vector of BLUEs; *μ* represents the population mean with assumed flat prior distribution; **a** is the vector of additive genetic effects, which follows a normal distribution with **a**∼𝑁(0, **G**𝜎_a_^2^), 𝜎_a_^2^ is the additive genetic variance, and **G** is the additive (genomic) relationship matrix; **d** is the vector of nonadditive genetic effects, which follows a normal distribution with **d**∼𝑁(0, **I**𝜎_d_^2^), 𝜎_d_^2^ is the nonadditive genetic variance, and **I** is an identity matrix; and **Z_a_** and **Z_d_** are incidence matrix of **a** and **d**, respectively; and 𝒆 is the vector of random errors with 𝒆∼𝑁(0, **I**𝜎_e_^2^), and 𝜎_e_^2^ is the residual variance. The construction of the genomic relationship matrix (**G**) was done using the AGHmatrix package in R (Amadeu et al., 2016). This model was implemented by the Bayesian generalized linear regression (BGLR) package in the R software (Pérez & de los Campos, 2014), defining 12,000 iterations for the Markov chain Monte Carlo (MCMC) algorithms, a burn‐in period of 2000 MCMC cycles, and thin equals to 5 before saving samples from each. The narrow sense heritability (*h^2^*) was computed as the posterior mean: $\left(h^{2}\right)=\frac{\sigma_{a}^{2}}{\sigma_{a}^{2}+\sigma_{e}^{2}}$ and the broad sense heritability $\left(H^{2}\right)=\frac{\sigma_{a}^{2}+\sigma_{d}^{2}}{\sigma_{a}^{2}+\sigma_{d}^{2}+\sigma_{e}^{2}}\;.$

### 2.7 Genome‐wide association study

To identify genomic regions regulating bacterial wilt resistance, we conducted GWAS using the R package GWASpoly v.2.11, specifically developed for autopolyploid analysis (Rosyara et al., 2016), separately for leaf severity and stem necrosis in the Comb_popn. To account for population structure, we constructed a **Q** matrix based on the top five principal components and calculated kinship matrices (**K**) using the algorithm embedded within the GWASpoly package. We assessed associations between SNPs and phenotype changes using a linear mixed model (**Q** + **K**) as outlined by Rosyara et al. (2016). Four distinct genetic models: general, additive, simplex dominant, and duplex dominant, were explored. In the additive models, the SNP effect scaled proportionally to allele dosage, while the dominant models examined the dominance of either the reference or alternative allele. We applied an Adjusted Bonferroni correction with a significance level of 0.05 to determine the *p*‐value detection threshold. Finally, we evaluated associations between SNPs and phenotypes using quantile–quantile plots, displaying estimated −log10(*p*) values. Independent GWAS analyses were performed for each population, Popn‐I and Popn‐II, for both leaf severity and stem necrosis traits, using BLUEs to assess marker‐trait associations. This strategy was adopted to evaluate the stability and cross‐population applicability of these markers using various GS models.

### 2.8 Candidate gene mining

After conducting the GWAS, we focused on genomic regions within a 100 kb range upstream and downstream of significantly associated SNPs using the *V. corymbosum* ‘Draper’ reference genome to identify potential candidate genes. Subsequently, we used the corresponding protein sequences as queries for functional annotation via PANNZER (Törönen & Holm, 2022) and eggNOG mapper (Huerta‐Cepas et al., 2019). Candidate genes with functional descriptions and gene ontology functions linked to plant resistance or defense‐related genes were chosen, and their potential roles corroborated through a literature search.

### 2.9 Genomic selection

The genomic prediction models were fitted utilizing the BGLR methodology. This was implemented in the BGLR package in R (Pérez & de los Campos, 2014) with 12,000 iterations, 2000 burn‐ins, and 5 thinning to estimate the posterior means of the genomic estimated breeding values as follows:

$$\;y^{*}=1\mu +\mathrm{Zu}+e$$

where **y** denotes a vector of BLUEs, previously adjusted for year effects in the case of the combined dataset; *μ* represents the population mean; ***e*** is the vector of random errors with $e∼N(0,I\sigma_{e}^{2})$, where **I** is the identity matrix and $\sigma_{e}^{2}$ is the residual variance; and **u** is the vector of genetic effect, and its incidence matrix Z, with **u**∼𝑁(***0***, **G** $\sigma_{g}^{2}$), and $\sigma_{g}^{2}$ is the genetic variance. The implementation of genomic prediction models was carried out with additive and dominance gene action models with different assumptions of prior distribution of marker effect. For additive models, **u** = **a**, where **a** is a vector of random effects that follows a multivariate normal distribution **a**∼𝑁(***0***, **G_a_** $\sigma_{a}^{2}$), where **G_a_** is the realized genetic relationship computed with genome‐wide markers following VanRaden et al. (2009) methodology, assuming tetrasomic inheritance and $\sigma_{a}^{2}$ is the additive variance. For additive and dominance models, **u** = **a** + **d**, where **a** and **d** are different vectors of random effects that follow multivariate normal distributions **a**∼(***0***, **G_a_** $\sigma_{a}^{2}$) and **d**∼𝑁(***0***, **G_d_** $\sigma_{d}^{2}$), respectively. **G_d_** is the digenic dominance genomic relationship matrix, and $\sigma_{d}^{2}$ is the digenic dominance variance. **G_a_** and **G_d_** were both constructed using the AGHmatrix package in R (Amadeu et al., 2016). BayesB model followed the mathematical notation described in Amadeu et al. (2020), where the genotypic effect of individual *i* is described as follows:

$$\;u_{i}=\sum_{k=1}^{38,000}X_{a}\alpha_{k}$$

where X_α_ is the genotypic matrix parameterized for additive effects, and α_k_ is the additive effect of the *k*th SNP marker.

To complement genomic best linear unbiased prediction (GBLUP) and BayesB, we evaluated additional prediction approaches including penalized linear regression and machine learning (ML) methods. Penalized regression models, including Ridge and Elastic Net, retain linear relationships between markers and phenotype while applying coefficient shrinkage. ML approaches, including random forest (RF), support vector machine (SVM), and extreme gradient boosting (XGB), were evaluated alongside penalized regression models to assess their predictive ability (PA) in high‐dimensional genomic data (González‐Camacho et al., 2018; Heslot et al., 2012). SVM was implemented using the e1071 package (Meyer et al., 2019), RF with the randomForest package (Liaw & Wiener, 2002), XGB with the xgboost package (T. Chen & Guestrin, 2016), and penalized regression models with the glmnet package (Friedman et al., 2010) in R.

In addition to genome‐wide models, two GWAS‐based approaches were also evaluated that included a marker‐assisted selection (MAS) model, where only the significant GWAS markers were included as fixed effects in a multiple linear regression framework, and a GS *de novo* GWAS model, which integrates the BGLR approach with significant GWAS markers included as fixed‐effect covariates.

PA was assessed through 10‐fold cross‐validation (CV) for both leaf severity and stem necrosis traits, based on BLUEs estimated within each population and the combined data. Five predictive frameworks were used that include the following: Within Comb_popn:CV, where models were trained and tested using nonoverlapping folds from the combined population; Within Popn‐I:CV and Within Popn‐II:CV, where CV was conducted separately within each individual population; and two interpopulation settings, Interpopn: Popn‐I to predict Popn‐II and Interpopn: Popn‐II to predict Popn‐I, where models were trained on one population to predict outcomes in the other.

## 3 RESULTS

### 3.1 Phenotypic analysis

**FIGURE 2:**
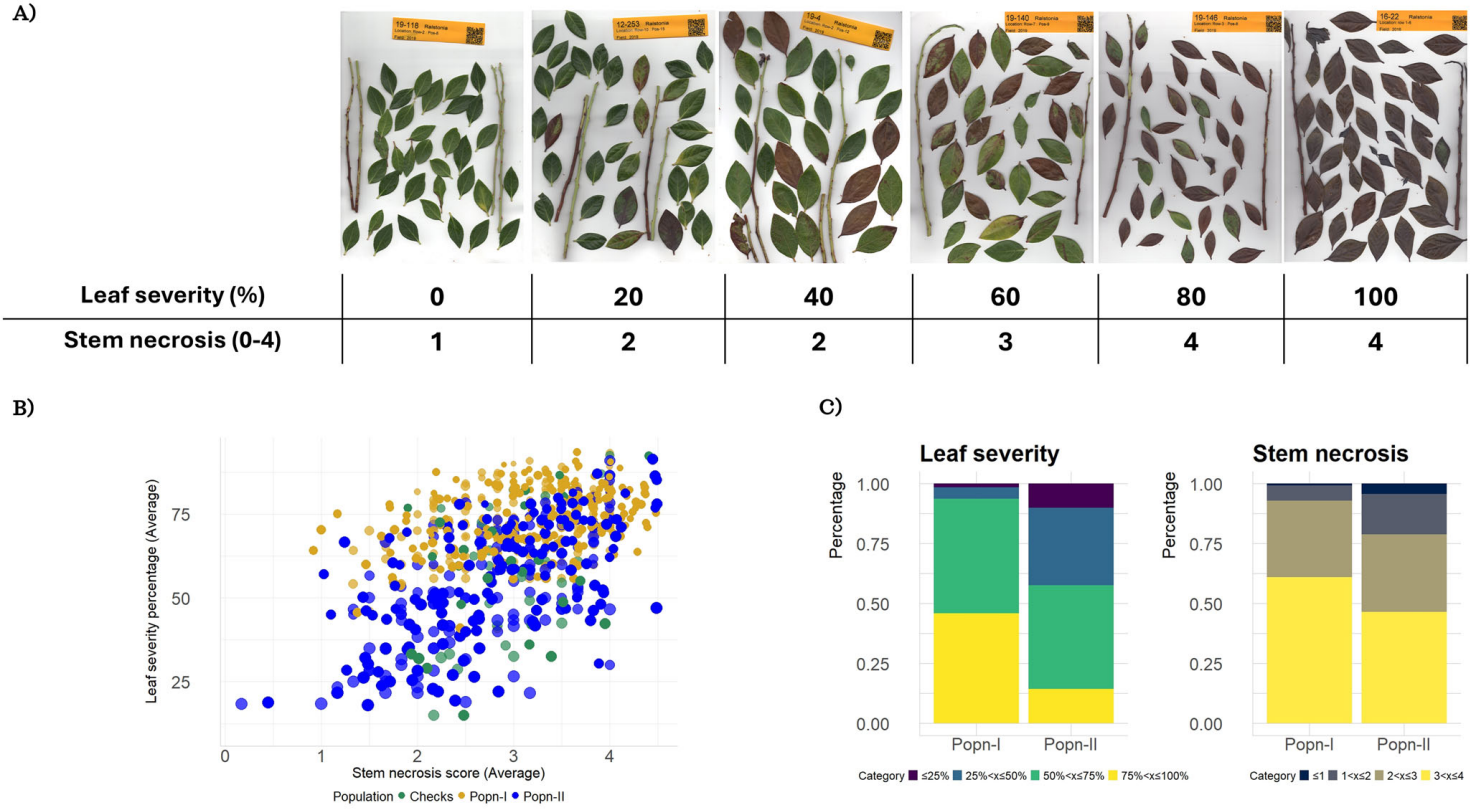
Phenotypic response exhibited by cuttings of blueberry genotypes against bacterial wilt following dip inoculation. (A) Illustration of the spectrum of disease responses from the visual assessment of leaf wilting severity and stem necrosis in various blueberry genotypes after inoculation with *R. pseudosolanacearum*. (B) Scatterplot showing the raw phenotypic distribution of leaf severity and stem necrosis scores across individual observations in two independent blueberry populations evaluated in separate years (Popn‐I in 2021; Popn‐II in 2023) after inoculation with *R. pseudosolanacearum*. Overlapping genotypes representing the 39 check individuals used for year‐to‐year correction are highlighted in green. (C) Stacked bar plot showing the percentage of total genotypes within each category for leaf severity and stem necrosis in two blueberry populations after inoculation with *R. pseudosolanacearum*.

We conducted visual assessments of disease severity following inoculation with *R. pseudosolanacearum* in two blueberry populations (Popn‐I and Popn‐II). The assessments were carried out separately for leaf severity and stem necrosis (Figure 2A). The correlation coefficient between leaf severity and stem necrosis was approximately 0.6. Our observations revealed broad phenotypic variation in disease severity across both leaf and stem assessments (Figure 2A,B). Typical symptoms of bacterial wilt on blueberries began to appear as early as 3 days after inoculation. While initial symptoms mostly appeared on the leaves and progressed with time after inoculation, there were several genotypes in both populations where stem necrosis initiated before visible leaf symptoms.

Average leaf severity exhibited a broad range in both populations, spanning from 8.75% to 97.5% in Popn‐I and from 5% to 100% in Popn‐II. Notably, a smaller proportion of genotypes in Popn‐I, approximately 1.6%, displayed an average leaf severity below 25%, compared to 10% of genotypes in Popn‐II, indicating a greater distribution of resistance in the latter (Figure 2C). The 39 check genotypes spanned this same range in both years, with the same genotype consistently anchoring the resistant extreme across both populations, confirming that the checks adequately represented the full spectrum of phenotypic diversity for year‐to‐year correction (Figure S2). For stem severity, the average scores ranged from 1 to 4 in Popn‐I and from 0.17 to 4 for Popn‐II. A smaller fraction, 0.8%, of the total genotypes in Popn‐I had average stem necrosis scores of ≤1, whereas in Popn‐II, a higher proportion, 4.2%, fell within this lower severity category, indicating a greater prevalence of lower necrosis scores in the latter (Figure 2C).

Upon combining both populations (Comb_popn) for the analysis, the variance attributed to nonadditive components was slightly higher than that attributed to additive components for both leaf severity and stem necrosis (Table 1). Furthermore, low narrow‐sense heritability (0.26 and 0.20) and moderate broad‐sense heritability (0.53 and 0.48) were observed for leaf severity and stem necrosis, respectively.

**TABLE 1:** Estimates of additive (*σ* ^2^_a_) and nonadditive (*σ* ^2^_d_) variance components, and narrow‐sense (*h*2) and broad‐sense (*H*2) heritabilities with their standard deviation (SD) in the combined blueberry population (Comb_popn) for bacterial wilt resistance symptoms.

Trait	*σ* ^2^_a _± SD	*σ* ^2^_d_ ± SD	*h*2 ± SD	*H*2 ± SD
Leaf severity	39.20 ± 12.80	41.40 ± 15.44	0.26 ± 0.08	0.53 ± 0.12
Stem necrosis	0.11 ± 0.03	0.16 ± 0.06	0.20 ± 0.06	0.48 ± 0.12

### 3.2 Genome‐wide association analysis

**FIGURE 3:**
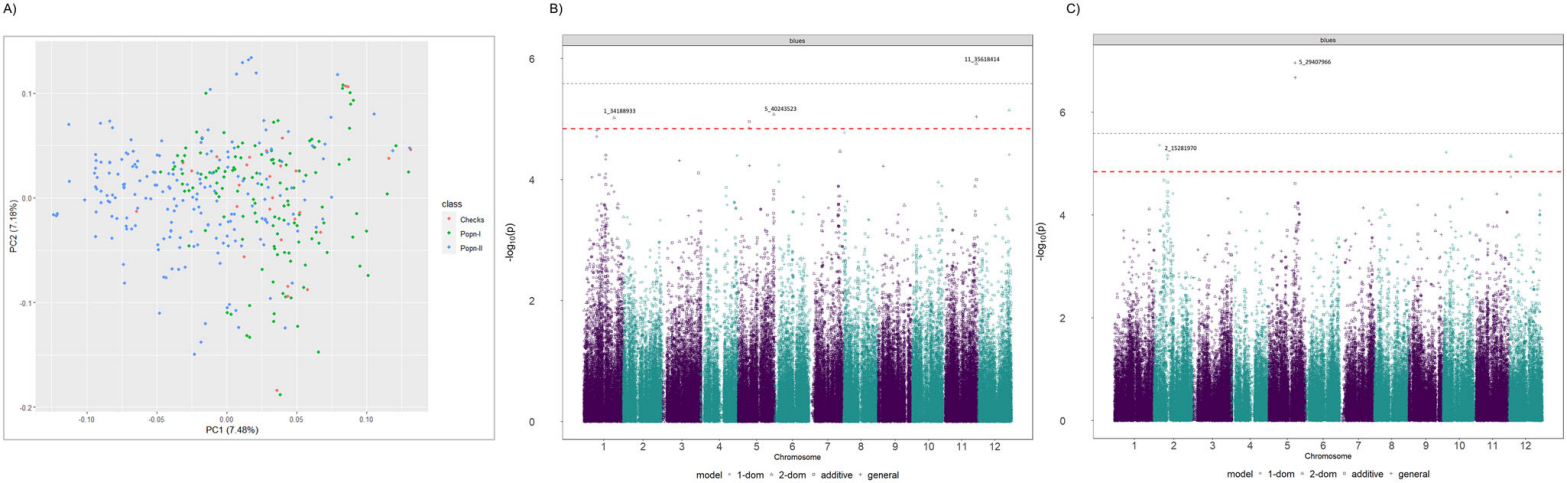
Population structure and genome‐wide association analysis for bacterial wilt resistance in blueberry. (A) Principal component analyses (PCA) of the two blueberry populations used in this study (Popn‐I and Popn‐II), comprising a total of 401 genotypes. PCA was performed using the single‐nucleotide polymorphism (SNP)‐based genomic relationship matrix. Each point represents an individual in the hyperspace defined by the eigenvectors of the first and second principal components. (B and C) Manhattan plots for bacterial wilt resistance traits with several gene action models in the blueberry population (Comb_popn) for (B) leaf severity symptoms and (C) stem necrosis symptoms, post inoculation with *R. pseudosolanacearum*. A linear mixed model with corrections for population structure and cryptic relatedness was used to compute the *p*‐values. An adjusted Bonferroni correction at *α* = 0.05 was used to establish the *p‐value* detection threshold for statistical significance. The black dashed line represents the most stringent genome‐wide threshold across all models tested, and the red dashed line indicates the model‐specific threshold for the 2‐dom‐alt gene‐action model.

A total of 38,379 SNPs, distributed across the 12 haploid blueberry chromosome‐scaled scaffolds, were independently tested for association with bacterial wilt resistance traits employing a GWAS approach (Figure S3). Population structure was assessed using principal component analysis based on the genomic relationship matrix, revealing overlapping yet distinguishable clusters corresponding to Popn‐I and Popn‐II (Figure 3A). When both populations were merged after correcting for the year‐effect (Comb_popn), we observed three significant associations in chromosomes 1, 5, and 11 for leaf severity, explaining 4.6%, 4.0%, and 4.4% of the phenotypic variance, respectively (Figure 3B, Figure S4A, Table 2). For stem necrosis, two significant associations were identified on chromosomes 2 and 5, explaining 5.3% and 7.4% of the phenotypic variance, respectively (Figure 3C, Figure S4B, Table 2). All identified markers were significant under the duplex‐dominant model, except marker 7_29407966, identified for stem necrosis.

**TABLE 2:** Significant single‐nucleotide polymorphisms (SNPs) associated to bacterial wilt in the combined blueberry population (Comb_popn) for two traits: leaf severity and stem necrosis. The corresponding SNP location, *p*‐value, minor allele frequency (MAF), effect, score (i.e., −log10(*p*‐value)), percentage of variance explained (PVE), and reference (Ref) and alternative (Alt) allele are reported.

Trait	Chr	Position	Model	*p*‐value	MAF	Effect	Threshold	Score	PVE	Ref/Alt
Leaf severity	1	34188933	2‐dom‐alt	0.00	0.23	16.60	4.84	5.02	4.6	T/A
	5	40243523	2‐dom‐alt	0.00	0.15	20.41	4.84	5.08	4.0	A/G
	11	35618414	2‐dom‐alt	0.00	0.06	−16.75	4.84	5.92	4.4	A/G
Stem necrosis	2	15281970	2‐dom‐alt	0.00	0.39	−0.34	4.84	5.16	5.3	A/C
	5	29407966	general	0.00	0.03	NA	5.58	6.96	7.4	C/T

To evaluate the potential of using markers identified within each population for GS in the other population, GWAS analyses were also conducted independently for each population, without pooling data across populations. In Popn‐I, GWAS for leaf severity identified nine significant associations across eight chromosomes, with markers 34188933 on chromosome 1 and 35618414 on chromosome 11, which were also detected in Comb_popn (Figures S5A and S6A, Table S1). In contrast, GWAS for Popn‐II detected a single significant marker on chromosome 8 for leaf severity (Figures S7A and S8A, Table S2). Similarly, an association on chromosome 11 was detected for stem necrosis (Figures S7B and S8B, Table S2). For stem necrosis, Popn‐I revealed marker 15281970 on chromosome 2, which was also detected in the Comb_popn analysis (Figure S5B and S6B, Table S1).

For gene mining, only markers identified from the Comb_popn analysis were considered, excluding population‐specific markers. Population‐specific markers were retained for genomic prediction to ensure their relevance in the overall model, and markers consistently identified in both individual and combined analyses were further tested individually and jointly for their PA.

### 3.3 Candidate gene mining

The putative protein‐coding genes located within a 100‐kb region upstream and downstream of significant SNPs identified for leaf severity and stem necrosis were extracted from the reference genome. Our analysis revealed the presence of 80 genes within the specified genomic regions (Table S3), and the candidate genes with the highest potential to be involved in regulating bacterial wilt resistance are presented in Table 3 and detailed in Table S3. Specifically, we identified genes encoding a putative pentatricopeptide repeat (PPR)‐containing protein, a serine‐threonine protein kinase, and an MYB‐related protein as potential candidates for regulating leaf severity, while genes encoding for mildew resistance locus protein and proteins involved in polysaccharide biosynthesis (xyloglucan and galactomannan) were identified as potential candidates regulating stem necrosis. Their potential role in plant defense is further explored in Section 4.

**TABLE 3:** Potential candidate genes related to bacterial wilt resistance that are within ± 100 kb of significant single‐nucleotide polymorphisms (SNPs) detected for leaf severity and stem necrosis in southern highbush blueberry population (Comb_popn).

Trait	Chr	Position	Gene	Gene description
Leaf severity	1	34188933	*VaccDscaff1‐processed‐gene‐342.6‐mRNA‐1*	Callose synthase
			VaccDscaff1‐augustus‐gene‐342.31‐mRNA‐1	Serine‐threonine protein kinase
	5	40243523	VaccDscaff7‐processed‐gene‐403.16‐mRNA‐1	MYB‐related protein
	11	35618414	*VaccDscaff21‐augustus‐gene‐355.27‐mrna‐1*	Pentatricopeptide repeat‐containing protein
			*VaccDscaff21‐augustus‐gene‐356.22‐mrna‐1*	bHLH‐like transcription factor
Stem necrosis	2	15281970	*VaccDscaff2‐snap‐gene‐153.40‐mRNA‐1*	MLO protein (Mildew resistance locus O)
	5	29407966	*VaccDscaff7‐augustus‐gene‐293.20‐mrna‐1*	Galactomannan galactosyltransferase
			*VaccDscaff7‐processed‐gene‐294.6‐mrna‐1*	Xyloglucan 6‐xylosyltransferase

### 3.4 Genomic selection

**FIGURE 4:**
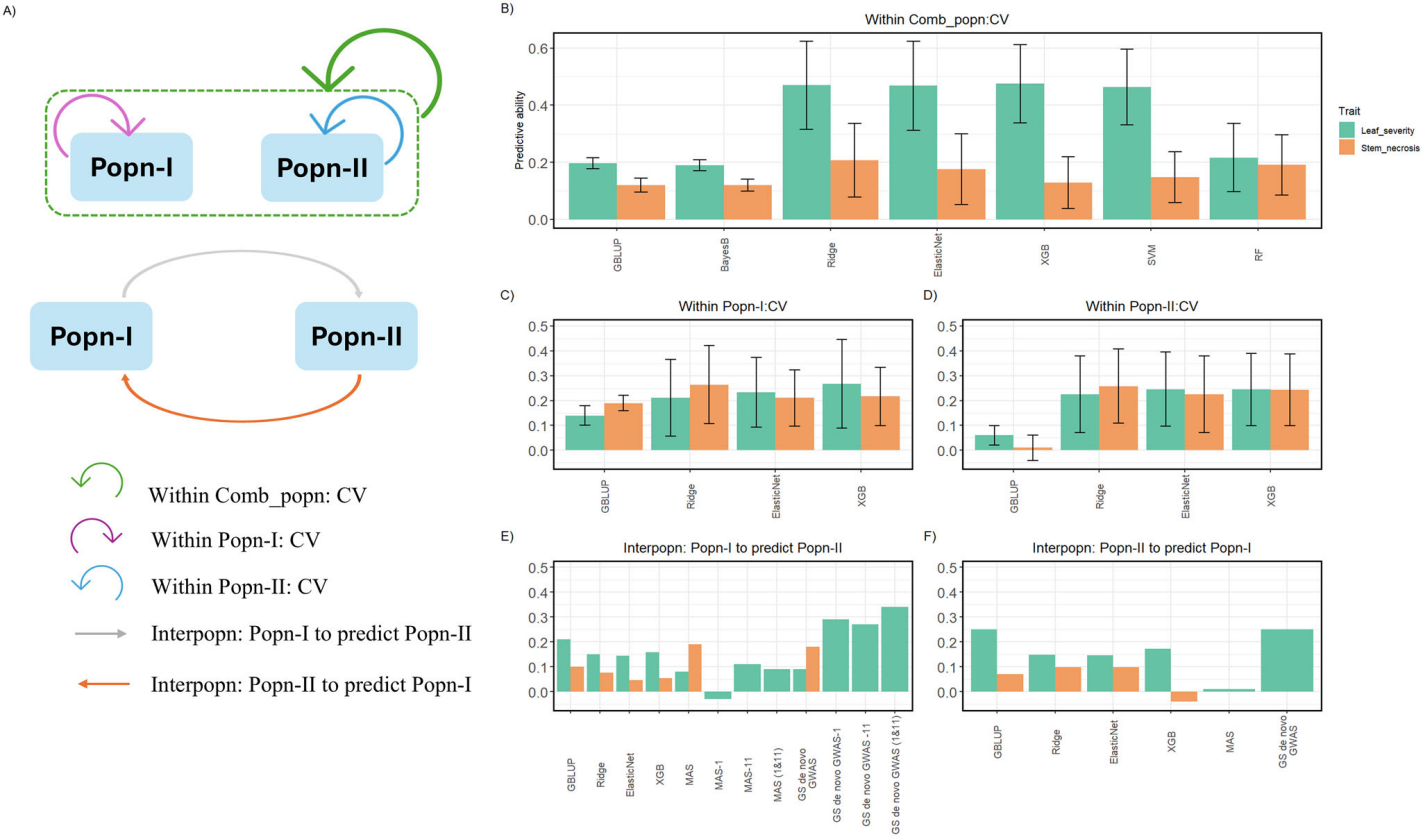
Genomic prediction for bacterial wilt resistance traits in blueberry. (A) The five genomic prediction frameworks evaluated in the study. Predictive ability for leaf severity and stem necrosis symptoms under each framework: (B) Within Comb_popn:CV (CV, cross‐validation), (C) Within Popn‐I:CV, (D) Within Popn‐II:CV, (E) Interpopn: Popn‐I to predict Popn‐II, and (F) Interpopn: Popn‐II to predict Popn‐I. Error bars in panels B–D represent the standard deviation across 10‐fold CV replicates. Panels E and F represent single interpopulation predictions and therefore do not include error bars.

We assessed the feasibility and effectiveness of GS for breeding bacterial‐wilt‐resistant blueberries, using five predictive frameworks that include three within‐population CV frameworks (i.e., Comb_popn, Popn‐I, and Popn‐II) and two interpopulation predictions (Popn‐I to predict Popn‐II and vice versa) (Figure 4A).

Within‐population predictions using 10‐fold CV showed that GBLUP (additive model) achieved PAs for leaf severity ranging from 0.06 in Popn‐II (Figure 4D) to 0.14 in Popn‐I (Figure 4C), increasing to 0.197 in the Comb_popn (Figure 4B). For stem necrosis, GBLUP PAs were 0.19 in Popn‐I (Figure 4C), 0.01 in Popn‐II (Figure 4D), and 0.12 for the Comb_popn (Figure 4B). Including dominance effects (GBLUP_add+dom) or the BayesB model did not markedly improve PA in any setting (Table S4). Penalized and ML models like XGM, SVM, Ridge, and Elastic Net consistently outperformed GBLUP and BayesB across all three within‐population frameworks, with highest PAs observed in Comb_popn (up to 0.48 for leaf severity and 0.27 for stem necrosis; Figure 4B–D, Table S4). Notably, Popn‐II, where GBLUP performed poorly, saw considerable gains from these models (Figure 4D).

In interpopulation predictions, where models were trained on one population and tested on the other, GBLUP yielded PAs of 0.21 and 0.25 for leaf severity when predicting Popn‐II from Popn‐I and vice versa, respectively (Figure 4E,F and Table S4). For stem necrosis, the corresponding values were 0.10 and 0.07. Penalized and ML models showed reduced performance in interpopulation settings, often falling below GBLUP (Figure 4E,F and Table S4). Several markers identified in Comb_popn were also present in the independent Popn‐I. In addition to testing the full marker set, we evaluated these shared markers individually and jointly under MAS and GS *de novo* GWAS models (Figure 4E,F).

For leaf severity, MAS using GWAS‐identified markers from Popn‐I to predict Popn‐II yielded a PA of 0.08, whereas the reverse (Popn‐II to predict Popn‐I) gave a much lower PA of 0.01. Substituting the full identified marker set with a single marker (34188933 on chromosome 1) further reduced the PA, while another marker substitution (35618414 on chromosome 11) increased the PA for Popn‐II to 0.11 (∼22% improvement). For stem necrosis, MAS with marker (marker 2_15281970) from Popn‐I to predict Popn‐II achieved a PA of 0.19. Reciprocal prediction for stem necrosis was not possible due to low allele frequencies (Figure 4F).

Among all models tested within the interpopulation framework, GS *de novo* GWAS models, combining GWAS‐identified markers as fixed effects with BGLR, most often delivered the highest PAs (Figure 4E,F). For stem necrosis, incorporating marker 2_15281970 identified in Popn‐I as a fixed effect when predicting Popn‐II achieved a PA of 0.18. For leaf severity, using all GWAS‐identified markers from Popn‐I to predict Popn‐II gave a PA of 0.09; however, when markers were evaluated individually, marker 35618414 on chromosome 11 alone improved the PA to 0.27, and marker 34188933 on chromosome 1 alone improved it to 0.29, with both markers jointly achieving the highest PA of 0.34. In the reverse direction, using marker from Popn‐II to predict Popn‐I yielded a PA of 0.25. These results underscore the strong predictive contribution of markers 35618414 and 34188933 for leaf severity, and marker 15281970 for stem necrosis, across populations.

## 4 DISCUSSION

The development of bacterial‐wilt‐resistant blueberries hinges on the identification of resistance sources and the comprehensive understanding of the genetic basis governing variability of disease severity within breeding populations. Despite its importance, the genetic basis of resistance to bacterial wilt caused by RSSC in blueberries remains under‐explored. In response to this knowledge gap, our study developed an assay to evaluate the response of SHB genotypes to *R. pseudosolanacearum* inoculation within a breeding program. Additionally, we conducted GWAS to elucidate the genetic variability associated with bacterial wilt resistance, identify molecular markers, and pinpoint candidate genes possibly linked to this resistance. Furthermore, genomic prediction analysis was employed to evaluate the practicality of genomic‐assisted selection for wilt‐resistant genotypes in the blueberry breeding program.

### 4.1 Phenotyping bacterial wilt resistance in blueberries

Investigation of genetic mechanisms regulating resistance requires initial screening of a large cohort of individuals (Sniezko & Nelson, 2022). Resistance can be rare and, therefore, screening of hundreds of individuals may be necessary to identify resistant sources for inclusion in breeding programs. Developing an efficient, high‐throughput screening protocol capable of testing hundreds of selections is crucial for breeding programs (Sniezko & Nelson, 2022). A previously reported method in blueberries involves cutting the stem near the soil surface and pipetting bacterial suspension into the wound (Conner et al., 2022). While effective, this approach can be resource‐intensive due to the long seedling maturation period and cumbersome when scaling to hundreds of selections.

Therefore, in this study, we developed an alternative assay using detached stem cuttings from two blueberry populations, inoculated with *R. pseudosolanacearum*, and evaluated for leaf severity and stem necrosis. This assay was conceptually informed by similar continuous immersion methods in cucurbits and potato (Freeman, 1993; Freeman & Rodriguez, 1992; Lapwood & Read, 1986). Continuous inoculum exposure ensured uniform disease pressure, minimized variability, and enabled high‐throughput screening. Unlike brief dip methods, this approach avoided missed exposures and allowed rapid visual confirmation of inoculation integrity. It also provided a stringent, field‐relevant assay reflective of the chronic soilborne nature of RSSC (Harmon et al., 2016), supporting reliable identification of resistant genotypes. While the detached stem assay lacks whole‐plant physiological responses and may introduce variability due to slight differences in stem maturity or post‐excision effects (Pettitt et al., 2011), it offers advantages such as rapid, high‐throughput screening and preservation of germplasm (Miller‐Butler et al., 2018). This approach enabled simultaneous evaluation of hundreds of advanced blueberry selections, with results attainable within 2 weeks. Furthermore, it is nondestructive to the source plant and can be conducted throughout the active growth period, eliminating the need to generate seedlings solely for bacterial wilt screening.

In our screening, we included four *Vaccinium* cultivars‐Emerald, Indigocrisp, Arcadia, and Ochlockonee‐as controls (both positive and negative). Across both screening rounds, Arcadia and Ochlockonee consistently displayed their expected levels of susceptibility and resistance, mirroring the outcomes reported by Conner et al. (2022) using the same phylotype I isolate P824. Although no genotype in our study, including Ochlockonee, exhibited the complete immunity reported by Conner et al. (2022), this likely reflects our assay's continuous inoculum exposure and the greater disease pressure it imposes compared to the methodology they adopted. Despite differences in assay format and scoring scales, the relative magnitude of phenotypic variation was preserved. On the other hand, Emerald produced a moderately resistant response while Indigocrisp exhibited susceptibility consistent with field performance. All these observations demonstrate that our protocol, although unique to other bacterial wilt screening protocols in blueberries, robustly captures the full spectrum of bacterial wilt resistance in SHB.

### 4.2 Genetic parameters and heritability

This study revealed broad phenotypic variation in leaf severity and stem necrosis, underscoring the presence of genetic variability within our studied population. The magnitude of nonadditive variance components was slightly higher than the magnitude of additive variance components for both leaf severity and stem necrosis in this study. Similarly, a study on resistance of tetraploid potatoes to *Ralstonia* also emphasized the importance of nonadditive effects in addition to the additive effects in this crop (Tung, 1992). The narrow‐sense and broad‐sense heritability estimates in our study were in the range of 0.2 and 0.5, respectively, which also aligned within the reported range of 0.12 and 0.65 for wilt‐disease index in tetraploid potato (Tung, 1992). These findings suggest the potential success of clonal selection in developing resistant blueberry cultivars that capture all genetic variances, as dominance effects can be heritable in polyploids unlike in diploid species (Amadeu et al., 2020).

Genetic correlations across years were estimated at 0.85 for leaf severity and 0.34 for stem necrosis. The substantially higher correlation observed for leaf severity suggests greater trait stability across years, whereas stem necrosis exhibited lower consistency across years, suggesting that stem necrosis may be more sensitive to year‐to‐year variation than leaf severity. This pattern was also reflected in the genomic analyses, where leaf severity showed more consistent marker associations and generally stronger predictive performance than stem necrosis. Together, these findings suggest that leaf severity may represent a more stable phenotype for investigating the genetic basis of bacterial wilt resistance and for implementing genomic‐assisted selection in blueberries.

### 4.3 GWAS of bacterial wilt resistance

Genomic studies on the genetic architecture of bacterial wilt resistance in plants have primarily utilized QTL mapping methods in crops like pepper (Du et al., 2019), tomato (Shin et al., 2020), potato (Habe et al., 2019), and tobacco (Drake‐Stowe et al., 2017). Recently, the use of GWAS has also been reported in common bean (Zia et al., 2022), Arabidopsis (Aoun et al., 2017), pepper (J.‐H. Lee et al., 2024), and tomato (Nguyen et al., 2021).

Finding that nonadditive variance exceeded additive variance for leaf severity and stem necrosis prompted us to explore GWAS models incorporating both effects in this study. GWAS revealed a total of five significant associations spanning different chromosomes, each one contributing to a small portion of the phenotypic variance for bacterial wilt leaf severity and stem necrosis. These findings align with the polygenic nature of bacterial wilt resistance observed in tobacco, tomato, and potato (Drake‐Stowe et al., 2017; Thoquet, 1996; Wang et al., 2000, 2013), where numerous broad‐spectrum QTLs have been mapped without identifying major resistance loci.

### 4.4 Candidate genes for bacterial wilt resistance

We next performed gene mining around significant GWAS hits to identify potential functional candidates for bacterial wilt resistance. RSSC enters plants via root openings or wounds, colonizes the stele, and spreads through xylem vessels (Hikichi et al., 2007). Early infection involves cell wall‐degrading enzymes and secretion of effector proteins, triggering plant defense responses that alter cell structures and metabolic networks (H. Shi et al., 2023). Accumulation of bacterial cells and exopolysaccharide in the xylem eventually blocks sap flow, leading to wilting (Hikichi et al., 2007). Because the plant cell wall is both a physical barrier and a primary target for RSSC (Kongala & Kondreddy, 2023), genes influencing its integrity are critical for resistance.

For stem necrosis, marker 5_29407966 co‐localized with two candidate genes encoding for enzymes in the biosynthesis of polysaccharides xyloglucan and galactomannan. Xyloglucan acts as a defense elicitor (Claverie et al., 2018; Y. B. Park & Cosgrove, 2015) and is associated with resistance responses in pepper (Hwang et al., 2011), tobacco (Pan et al., 2021), and peanut (Y. Chen et al., 2014) following *R. solanacearum* infection (Claverie et al., 2018; Y. B. Park & Cosgrove, 2015). Galactomannan contributes to cell wall structure and water retention, with changes in its composition linked to stress responses and vascular occlusion (Edwards et al., 1999; Sharma et al., 2021).

Near marker 1_34188933 identified for leaf severity, we detected a gene encoding a serine/threonine‐protein kinase (Pkinase), a class of receptor‐like kinases that mediate pathogen recognition and defense signaling. In Solanaceae, serine/threonine kinases are rapidly induced during *R. solanacearum* infection, triggering defense gene activation, reactive oxygen species (ROS) bursts, and cell wall reinforcement (Lavale et al., 2022; L. Shi et al., 2024). The same locus contained a callose synthase (*FKS1_dom1*) gene, responsible for callose biosynthesis. Callose deposition is a well‐documented defense against vascular pathogens, restricting *R. solanacearum* movement within the xylem (H. Shi et al., 2023; Xue et al., 2025).

Marker 5_40243523 for leaf severity co‐localized with a gene encoding an MYB‐HB‐like transcription factor, known to regulate phenylpropanoid metabolism, lignin biosynthesis, and defense gene expression. MYB transcription factors enhance *R. solanacearum* resistance in tomato, eggplant, and potato (N. Chen et al., 2024; Z. Chen et al., 2025; Qiu et al., 2019).

Marker 11_35618414 for leaf severity was near a putative pentatricopeptide repeat (PPR) gene, previously implicated in anthracnose resistance in blueberry (Ghimire et al., 2025) and in *R. solanacearum* defense in potato via regulation of mitochondrial ROS homeostasis (S. Park et al., 2016). The same region also harbored a bHLH transcription factor gene, with homologs shown to mediate *R. solanacearum*‐triggered hormone signaling in pepper (Hussain et al., 2021) and to be differentially expressed in eucalyptus after infection (Xiaohui et al., 2022).

Finally, marker 2_15281970 for stem necrosis was located near a gene encoding an MLO (Mildew resistance locus O) protein, a well‐known susceptibility factor. *Mlo* genes regulate cell wall integrity and defense signaling, and loss‐of‐function alleles confer durable resistance to powdery mildew in crops such as barley (Büschges et al., 1997; Jørgensen, 1992) and tomato (Bai et al., 2008). Multiple studies have also demonstrated a connection between MLO family members and *R. solanacearum* interactions, with *Mlo* genes in tomato showing altered expression following infection and functional changes affecting pathogen colonization and wilt symptom development (Huang et al., 2024; J. Shi et al., 2020).

We recognize that the genes highlighted here may serve multiple functions beyond defense, and that other genomic regions likely contribute to bacterial wilt resistance. Nonetheless, the strong literature support for *Mlo*, Pkinase, *FKS1*, MYB‐HB, PPR, and bHLH, combined with their co‐localization with significant GWAS signals, makes these loci high‐priority candidates for functional validation in blueberry.

### 4.5 Genomic prediction and prediction frameworks

Genomic prediction has been utilized in blueberries for a range of traits, including fruit quality (Adunola et al., 2024; de Bem Oliveira et al., 2019; Ferrão et al., 2020), postharvest characteristics (Casorzo et al., 2024), and parthenocarpy‐related traits (Cromie et al., 2025), but its application to disease resistance, particularly for bacterial wilt, remains unexplored. Although RSSC has a broad host range, GS studies targeting resistance to this pathogen are limited even in other crop species. One study in diploid common beans reported a PA in the range of 0.37–0.46 for bacterial wilt (Zia et al., 2022).

In this study, we evaluated GS strategies across five predictive frameworks: (i) CV within the combined population, (ii) CV within Popn‐I, (iii) CV within Popn‐II, (iv) interpopulation prediction using Popn‐I to predict Popn‐II, and (v) the reverse interpopulation prediction. Models included the baseline genomic prediction approaches (GBLUP and BayesB), penalized regression methods (Ridge and Elastic Net), and ML approaches such as SVM, XGB, and RF, along with a GS *de novo* GWAS approach and MAS model using key GWAS‐identified markers.

Within the combined population framework, XGB, SVM, Ridge, and Elastic Net models consistently yielded higher PAs compared to GBLUP and BayesB for leaf severity by approximately 2.5 times. This aligns with prior work highlighting the ability of these models to emphasize informative marker effects while shrinking the contribution of less informative predictors in high‐dimensional genomic data (T. Chen & Guestrin, 2016; Ogutu et al., 2012). When CV was done within Popn‐I and Popn‐II, these models again exceeded GBLUP, further supporting their advantage over GBLUP in within‐population prediction settings. However, in interpopulation predictions, training in one population and predicting in another, these models exhibited a sharp decline in PA, often underperforming GBLUP. This is likely because these models are more sensitive to population structure, differences in allele frequencies, and LD patterns between training and testing sets (Heslot et al., 2012), and may also capture population‐ or year‐specific patterns that do not transfer well across populations. In contrast, GBLUP, which models genome‐wide additive effects through the genomic relationship matrix, captures a more stable and transferable genetic signal, making it inherently more robust in interpopulation settings (de los Campos et al., 2013). This interpretation is supported by the estimated genetic correlations across years, which were higher for leaf severity (0.85) than for stem necrosis (0.34). The higher correlation observed for leaf severity is consistent with its stronger performance in interpopulation prediction. However, the loci identified by GWAS individually explained only a small proportion of the phenotypic variance and were therefore sensitive to modest changes in their estimated effects across populations, contributing to inconsistency in marker associations across populations. Similarly, BayesB showed performance comparable to GBLUP across scenarios, consistent with findings by Amadeu et al. (2020).

Interestingly, when comparing the performance of penalized and ML models across the Comb_popn and individual subpopulations, a clear pattern emerged. These models achieved high PA within Comb_popn, exceeding their performance within either subpopulation (Popn‐I or Popn‐II), benefitting from increased sample size and broader genetic variation. This finding aligns with previous studies in blueberries that suggest that increasing training population size enhances PA, particularly for penalized and ML models, by capturing a more comprehensive spectrum of genetic variance and reducing overfitting (Adunola et al., 2024; de Bem Oliveira et al., 2020).

Although our GWAS revealed that dominance effects contributed substantially to trait variance, including dominance effects in GS models did not improve PA relative to additive‐only models. This result is consistent with studies in blueberry (Amadeu et al., 2020), wheat (Zhao et al., 2013), pine (de Almeida Filho et al., 2016), and citrus (Minamikawa et al., 2017), which have shown that dominance variance may be biologically important but contributes little to prediction accuracy in genome‐wide models. Furthermore, the broadly similar predictive performance observed between penalized linear models (Ridge and Elastic Net) and nonlinear ML approaches across several prediction frameworks suggests that additive genetic effects captured most of the predictive signal for bacterial wilt resistance in this population. As suggested by Amadeu et al. (2020), while dominance effects can be significant at the level of individual genes (Ferrão et al., 2018), they are often diluted when modeled across the genome, especially in datasets of limited size (Minamikawa et al., 2017; Zhao et al., 2013).

MAS models based on significant GWAS hits, particularly marker 11_35618414 on chromosome 11, demonstrated strong predictive power for leaf severity. In Popn‐II, using this single marker in MAS increased PA by ∼22% relative to using the full GWAS marker set. This observation is consistent with studies in blueberry where key markers disproportionately influenced trait prediction, as shown for parthenocarpy‐related traits (Cromie et al., 2025).

The GS *de novo* GWAS framework, which integrates GWAS‐derived markers as fixed effects while modeling the remaining genome‐wide markers as random effects, produced the highest PAs for both traits. This finding aligns with work in several crop species, where anchoring models to biologically relevant loci significantly improved prediction (Bernardo, 2014; Ferrão et al., 2020; Rice & Lipka, 2019). For leaf severity, markers 11_35618414 and 1_34188933 again proved critical, achieving PAs of 0.27 and 0.29 individually, and 0.34 when combined. These markers were selected for both MAS and GS *de novo* GWAS models due to their consistent identification in Popn‐I and Comb_popn. These findings reinforce their reliability and their role in enhancing predictive performance for bacterial wilt resistance in blueberry breeding programs. GS *de novo* GWAS was not evaluated under within‐population CV framework to avoid circularity between marker discovery and model evaluation, which can inflate PA; instead, it was assessed in interpopulation scenarios only, aligning with its intended application in breeding. For polygenic traits, where small‐effect markers may be overlooked in conventional GWAS, GS *de novo* GWAS can more effectively capture their cumulative effects by fixing the significant loci while incorporating all genome‐wide markers as random effects compared to MAS (Bernardo, 2014; Ferrão et al., 2020). The success of the GS *de novo* GWAS framework emphasizes the importance of leveraging significant markers within predictive models.

These results demonstrate the complementary strengths of different GS approaches. Penalized regression and ML models are particularly effective within populations and under CV frameworks; GBLUP is more robust in interpopulation scenarios, and GS *de novo* GWAS offers the highest PA when trait‐associated loci are identified and show consistent effects across populations. These findings underscore the importance of tailoring model choice to genetic structure, dataset size, and the genetic architecture of the trait, while also highlighting the translational potential of GWAS‐anchored prediction in disease resistance breeding for perennial crops.

### 4.6 Breeding implications

Overall, our study highlights the broad genetic variability for resistance to bacterial wilt in blueberries, highlighting its potential for advancing breeding strategies. While narrow‐sense heritability estimates for the traits were low, moderate broad‐sense heritability values suggest the feasibility of achieving genetic gains through targeted breeding efforts, given the heritable dominance effects in polyploids. The identification of five small‐effect QTLs associated with resistance, along with genes implicated in host defense mechanisms, highlights opportunities for leveraging broad‐spectrum resistance. GS demonstrated utility in breeding programs despite moderate PAs across populations, with the identification of key markers, such as marker 1_34188933 and 11_35618414, illustrating the potential for integrating molecular tools to enhance selection precision. Future breeding efforts should focus on validating identified QTLs in independent populations and, given that our screening used only *R. pseudosolanacearum* phylotype I, sequevar 13, isolate P824, also against additional, genetically diverse *Ralstonia* strains to confirm their robustness and broad applicability. These findings provide a roadmap for incorporating genomic strategies into blueberry breeding programs and suggest broader applicability to other traits and crop species.

## 5 CONCLUSION

Our study aimed to uncover the genetic basis of bacterial wilt resistance caused by *R. pseudosolanacearum* in blueberries. We developed a high‐throughput screening protocol and assessed the response of 401 genotypes from two independent blueberry populations against this disease by measuring leaf severity and stem necrosis symptoms. We explored the genetic components of these traits and leveraged GWAS to pinpoint genomic regions associated with observed disease response. To assess their practical value, we incorporated GWAS‐identified markers into genomic prediction models and evaluated their PA for bacterial wilt resistance. We also identified potential candidate genes supported by literature, offering promising targets for functional validation. Together, these SNPs, candidate genes, and prediction results provide valuable resources for molecular breeding strategies aimed at enhancing bacterial wilt resistance in blueberries.

## AUTHOR CONTRIBUTIONS

**Lushan Ghimire**: Data curation; formal analysis; investigation; methodology; resources; validation; visualization; writing—original draft; writing—review and editing. **Norma Flor**: Investigation; methodology; writing—review and editing. **Paul Adunola**: Formal analysis; writing—review and editing. **Philip F. Harmon**: Conceptualization; investigation; methodology; supervision; writing—review and editing. **Felix Enciso‐Rodriguez**: Investigation; supervision; writing—review and editing. **Juliana Benevenuto**: Investigation; writing—review and editing. **Luis Felipe V. Ferrão**: Investigation; writing—review and editing. **Patricio R. Munoz**: Conceptualization; formal analysis; funding acquisition; investigation; methodology; project administration; writing—review and editing.

## CONFLICT OF INTEREST STATEMENT

The authors declare no conflicts of interest.

## Supporting information




**Figure S1**. Softwood cuttings of southern highbush blueberry population dipped continuously in the inoculum of *R. pseudosolanacearum* on a plastic trough and incubated inside a walk‐in growth chamber. Each trough represents one replicate in the experiment. The softwood cuttings in three beakers represent negative controls dipped in deionized water for each of the three replicates.
**Figure S2**. Scatterplots showing the year‐to‐year consistency of bacterial wilt disease for 39 overlapping check genotypes evaluated in 2021 (Popn‐I) and 2023 (Popn‐II) following inoculation with *R. pseudosolanacearum* for (A) Leaf severity and (B) Stem necrosis. r= Pearson correlation coefficient..
**Figure S3**. Distribution of 38,379 filtered single nucleotide polymorphisms (SNPs) in 1 Mb window size across the 12 blueberry chromosomes. The x‐axis represents the distance in base pairs.
**Figure S4**. Quantile‐quantile (Q‐Q) plots of GWAS results for bacterial wilt resistance with distinct gene action models in blueberry population (Comb_popn): A) Q‐Qplot for leaf severity and B) Q‐Qplot for stem necrosis. The observed association significance is plotted against the expected association significance for each SNP.
**Figure S5**. Manhattan plots for bacterial wilt resistance traits with several gene action models in blueberry population (Popn‐I) for A) leaf severity symptoms and, B) stem necrosis symptoms, post inoculation with *R. pseudosolanacearum*. A linear mixed model with corrections for population structure and cryptic relatedness was used to compute the *p*‐values. Adjusted Bonferroni correction, considering a genome‐wide significance level of 0.05 (dotted line), was used to establish a *p*‐value detection threshold for statistical significance.
**Figure S6**. Quantile‐quantile (Q‐Q) plots of GWAS results for bacterial wilt resistance with distinct gene action models in blueberry population (Popn‐I): A) Q‐Qplot for leaf severity and B) Q‐Qplot for stem necrosis. The observed association significance is plotted against the expected association significance for each SNP.
**Figure S7**. Manhattan plots for bacterial wilt resistance traits with several gene action models in blueberry population (Popn‐II) for A) leaf severity symptoms and, B) stem necrosis symptoms, post inoculation with *R. pseudosolanacearum*. A linear mixed model with corrections for population structure and cryptic relatedness was used to compute the *p*‐values. Adjusted Bonferroni correction, considering a genome‐wide significance level of 0.05 (dotted line), was used to establish a *p*‐value detection threshold for statistical significance.
**Figure S8**. Quantile‐quantile (Q‐Q) plots of GWAS results for bacterial wilt resistance with distinct gene action models in blueberry population (Popn‐II): A) Q‐Qplot for leaf severity and B) Q‐Qplot for stem necrosis. The observed association significance is plotted against the expected association significance for each SNP.
**Table S1**. Significant SNPs associated to bacterial wilt in the blueberry population (Popn‐I) for two traits: leaf severity and stem necrosis. The corresponding SNP location, p‐value, minor allele frequency (MAF), effect, score (i.e. ‐log10(*p*‐value)), percentage of variance explained (PVE), and reference (Ref) and alternative (Alt) allele are reported.
**Table S2**. Significant SNPs associated to bacterial wilt in the blueberry population (Popn‐II) for two traits: leaf severity and stem necrosis. The corresponding SNP location, p‐value, minor allele frequency (MAF), effect, score (i.e. ‐log10(*p*‐value)), percentage of variance explained (PVE), and reference (Ref) and alternative (Alt) allele are reported.
**Table S4**. Predictive abilities (PA) of genomic selection models across five evaluation frameworks for two bacterial wilt resistance traits: leaf severity and stem necrosis, in blueberry. Models evaluated include GBLUP, BayesB, ElasticNet, extreme gradient boosting (XGB), Support Vector Machine (SVM), Random Forest (RF), Ridge regression (Ridge), GS *de novo* GWAS, and marker‐assisted selection (MAS), under within‐ and inter‐ population cross‐validation settings. PA values are presented separately for each trait.

**Table S3**. Functional annotation of potential candidate genes located in genomic regions significantly associated with bacterial wilt resistance.

Supplementary Material

## Data Availability

The genomic and phenotypic data generated and analyzed during this study are available as Supporting Information.
