# Supplementary material for: Genetic insights into bacterial wilt resistance using genomic prediction and association mapping in blueberry
Source: Plant Genome. 2026 Aug 20;19(3):e70283. doi: 10.1002/tpg2.70283 (PMC13493699; doi:10.1002/tpg2.70283)
Supplement: Supplementary file 1 [file TPG2-19-e70283-s003.docx]

**SUPPLEMENTAL FIGURES**

**GENOME-WIDE ASSOCIATION STUDY AND GENOMIC SELECTION FOR BACTERIAL WILT RESISTANCE IN BLUEBERRIES**

**Figure S1**. Softwood cuttings of southern highbush blueberry population dipped continuously in the inoculum of *R. pseudosolanacearum* on a plastic trough and incubated inside a walk-in growth chamber. Each trough represents one replicate in the experiment. The softwood cuttings in three beakers represent negative controls dipped in deionized water for each of the three replicates.

Figure S2. Scatterplots showing the year-to-year consistency of bacterial wilt disease for 39 overlapping check genotypes evaluated in 2021 (Popn-I) and 2023 (Popn-II) following inoculation with *R. pseudosolanacearum* for (A) Leaf severity and (B) Stem necrosis. r = Pearson correlation coefficient.

**Figure S3**. Distribution of 38,379 filtered single nucleotide polymorphisms (SNPs) in 1 Mb window size across the 12 blueberry chromosomes. The x-axis represents the distance in base pairs.

A) B)

**Figure S4**. Quantile-quantile (Q-Q) plots of GWAS results for bacterial wilt resistance with distinct gene action models in blueberry population (Comb_popn): A) Q-Qplot for leaf severity and B) Q-Qplot for stem necrosis. The observed association significance is plotted against the expected association significance for each SNP.

A) B)

**Figure S5**. Manhattan plots for bacterial wilt resistance traits with several gene action models in blueberry population (Popn-I) for A) leaf severity symptoms and, B) stem necrosis symptoms, post inoculation with *R. pseudosolanacearum.* A linear mixed model with corrections for population structure and cryptic relatedness was used to compute the *p*-values. Adjusted Bonferroni correction, considering a genome-wide significance level of 0.05 (dotted line), was used to establish a *p*-value detection threshold for statistical significance.

A) B)

**Figure S6**. Quantile-quantile (Q-Q) plots of GWAS results for bacterial wilt resistance with distinct gene action models in blueberry population (Popn-I): A) Q-Qplot for leaf severity and B) Q-Qplot for stem necrosis. The observed association significance is plotted against the expected association significance for each SNP.

A) B)

**Figure S7**. Manhattan plots for bacterial wilt resistance traits with several gene action models in blueberry population (Popn-II) for A) leaf severity symptoms and, B) stem necrosis symptoms, post inoculation with *R. pseudosolanacearum.* A linear mixed model with corrections for population structure and cryptic relatedness was used to compute the *p*-values. Adjusted Bonferroni correction, considering a genome-wide significance level of 0.05 (dotted line), was used to establish a *p*-value detection threshold for statistical significance.

A)	B)

**Figure S8**. Quantile-quantile (Q-Q) plots of GWAS results for bacterial wilt resistance with distinct gene action models in blueberry population (Popn-II): A) Q-Qplot for leaf severity and B) Q-Qplot for stem necrosis. The observed association significance is plotted against the expected association significance for each SNP.

**Table S1**. Significant SNPs associated to bacterial wilt in the blueberry population (Popn-I) for two traits: leaf severity and stem necrosis. The corresponding SNP location, p-value, minor allele frequency (MAF), effect, score (i.e. -log10(*p*-value)), percentage of variance explained (PVE), and reference (Ref) and alternative (Alt) allele are reported.

| Trait | Chr | Position | Model | p-value | MAF | Effect | Threshold | Score | PVE | Ref/Alt |
| --- | --- | --- | --- | --- | --- | --- | --- | --- | --- | --- |
| Leaf severity | 1 | 34188933 | 2-dom-alt | 0.000 | 0.25 | 20.17 | 4.72 | 5.88 | 6.8 | T/A |
|  | 3 | 18374599 | general | 0.283 | 0.14 | NA | 5.53 | 5.79 | 1.5 | A/G |
|  | 4 | 40816119 | general | 0.214 | 0.03 | NA | 5.53 | 6.38 | 1.2 | G/A |
|  | 6 | 11703999 | general | 0.002 | 0.09 | NA | 5.53 | 7.54 | 4.8 | G/A |
|  | 6 | 19728311 | general | 0.762 | 0.05 | NA | 5.53 | 5.58 | 0.2 | C/A |
|  | 8 | 16215665 | additive | 0.00 | 0.11 | -5.52 | 5.53 | 5.59 | 7.2 | C/T |
|  | 9 | 5781233 | general | 0.643 | 0.03 | NA | 5.53 | 5.64 | 0.4 | C/A |
|  | 11 | 35618414 | 2-dom-alt | 0.00 | 0.08 | -19.79 | 4.72 | 5.88 | 9.5 | A/G |
|  | 12 | 12836277 | general | 0.805 | 0.03 | NA | 5.53 | 5.59 | 0.2 | T/C |
| Stem necrosis | 2 | 15281970 | additive | 0.00 | 0.37 | -0.26 | 5.53 | 5.88 | 6.7 | A/C |

**Table S2**. Significant SNPs associated to bacterial wilt in the blueberry population (Popn-II) for two traits: leaf severity and stem necrosis. The corresponding SNP location, p-value, minor allele frequency (MAF), effect, score (i.e. -log10(*p*-value)), percentage of variance explained (PVE), and reference (Ref) and alternative (Alt) allele are reported.

| Trait | Chr | Position | Model | p-value | MAF | Effect | Threshold | Score | PVE | Ref/Alt |
| --- | --- | --- | --- | --- | --- | --- | --- | --- | --- | --- |
| Leaf severity | 8 | 1099350 | 1-dom-ref | 0.00 | 0.17 | 10.40 | 5.54 | 5.72 | 11.4 | A/C |
| Stem necrosis | 11 | 24033834 | General, 2-dom-ref | 0.14, 1.00 | 0.01 | NA, 1.66 | 5.57, 5.20 | 5.59,5.26 | 2.8,  0.00 | G/A |

**Table S3**. Functional annotation of potential candidate genes located in genomic regions significantly associated with bacterial wilt resistance.

Note: This table is presented in a separate excel file labelled **Table S3**.

**Table S4**. Predictive abilities (PA) of genomic selection models across five evaluation frameworks for two bacterial wilt resistance traits: leaf severity and stem necrosis, in blueberry. Models evaluated include GBLUP, BayesB, ElasticNet, XGBoost (XGB), Support Vector Machine (SVM), Random Forest (RF), Ridge regression (Ridge), GS *de novo* GWAS, and marker-assisted selection (MAS), under within- and inter- population cross-validation settings. PA values are presented separately for each trait.

| GS framework | Model | Leaf severity | Stem necrosis |
| --- | --- | --- | --- |
| Within Comb_popn:CV | GBLUP | 0.197 | 0.12 |
| Within Comb_popn:CV | GBLUP_add+dom | 0.191 | 0.12 |
| Within Comb_popn:CV | BayesB | 0.19 | 0.12 |
| Within Comb_popn:CV | Ridge | 0.47 | 0.21 |
| Within Comb_popn:CV | ElasticNet | 0.47 | 0.18 |
| Within Comb_popn:CV | XGB | 0.48 | 0.13 |
| Within Comb_popn:CV | SVM | 0.46 | 0.15 |
| Within Comb_popn:CV | RF | 0.22 | 0.19 |
| Within Popn-I:CV | GBLUP | 0.14 | 0.19 |
| Within Popn-I:CV | GBLUP_add+dom | 0.13 | 0.19 |
| Within Popn-I:CV | BayesB | 0.14 | 0.18 |
| Within Popn-I:CV | Ridge | 0.21 | 0.26 |
| Within Popn-I:CV | ElasticNet | 0.23 | 0.21 |
| Within Popn-I:CV | XGB | 0.27 | 0.22 |
| Within Popn-I:CV | SVM | 0.24 | 0.27 |
| Within Popn-I:CV | RF | 0.24 | 0.25 |
| Within Popn-II:CV | GBLUP | 0.06 | 0.01 |
| Within Popn-II:CV | GBLUP_add+dom | 0.06 | 0.03 |
| Within Popn-II:CV | BayesB | 0.06 | 0.02 |
| Within Popn-II:CV | Ridge | 0.23 | 0.26 |
| Within Popn-II:CV | ElasticNet | 0.25 | 0.23 |
| Within Popn-II:CV | XGB | 0.24 | 0.24 |
| Within Popn-II:CV | SVM | 0.24 | 0.23 |
| Within Popn-II:CV | RF | 0.22 | 0.24 |
| Interpopn: Popn-I to predict Popn-II | GBLUP | 0.21 | 0.10 |
| Interpopn: Popn-I to predict Popn-II | Ridge | 0.15 | 0.08 |
| Interpopn: Popn-I to predict Popn-II | ElasticNet | 0.15 | 0.05 |
| Interpopn: Popn-I to predict Popn-II | XGB | 0.16 | 0.05 |
| Interpopn: Popn-I to predict Popn-II | SVM | 0.15 | 0.05 |
| Interpopn: Popn-I to predict Popn-II | RF | 0.11 | 0.11 |
| Interpopn: Popn-I to predict Popn-II | MAS | 0.08 | 0.19 |
| Interpopn: Popn-I to predict Popn-II | MAS-1 | -0.03 |  |
| Interpopn: Popn-I to predict Popn-II | MAS-11 | 0.11 |  |
| Interpopn: Popn-I to predict Popn-II | MAS (1&11) | 0.09 |  |
| Interpopn: Popn-I to predict Popn-II | GS *de novo* GWAS | 0.09 | 0.18 |
| Interpopn: Popn-I to predict Popn-II | GS *de novo* GWAS-1 | 0.29 |  |
| Interpopn: Popn-I to predict Popn-II | GS *de novo* GWAS -11 | 0.27 |  |
| Interpopn: Popn-I to predict Popn-II | GS *de novo* GWAS (1&11) | 0.34 |  |
| Interpopn: Popn-II to predict Popn-I | GBLUP | 0.25 | 0.07 |
| Interpopn: Popn-II to predict Popn-I | Ridge | 0.15 | 0.09 |
| Interpopn: Popn-II to predict Popn-I | ElasticNet | 0.15 | 0.09 |
| Interpopn: Popn-II to predict Popn-I | XGB | 0.17 | -0.04 |
| Interpopn: Popn-II to predict Popn-I | SVM | 0.15 | 0.10 |
| Interpopn: Popn-II to predict Popn-I | RF | 0.16 | 0.00 |
| Interpopn: Popn-II to predict Popn-I | MAS | 0.01 |  |
| Interpopn: Popn-II to predict Popn-I | GS de novo GWAS | 0.25 |  |

Note:

GBLUP = Genomic Best Linear Unbiased Prediction (additive effects only)

GBLUP_add+dom = GBLUP model including both additive and dominance effects

MAS-1 and GS de novo GWAS-1 = respective models using a single significant marker on Chromosome 1 identified for leaf severity in Popn-I and Comb_popn (marker: 1_34188933, position: 34,188,933bp)

MAS-11 and GS de novo GWAS-11 = respective models using a single significant marker on Chromosome 11 identified for leaf severity in Popn-I and Comb_popn (marker: 11_35618414, position: 35,618,414 bp)

MAS (1&11) and GS de novo GWAS (1 & 11) = respective models using both significant markers identified for leaf severity in Popn-I and Comb_popn (marker: 1_34188933 and marker: 11_35618414)
